# Hospitalized Cancer Patients with Opioid Management for Chemo-Induced Ulcerative Mucositis Lessens the Patients’ Overall Burden of Illness

**DOI:** 10.3390/ph18040536

**Published:** 2025-04-06

**Authors:** Minu Ponnamma Mohan, Joel B. Epstein, Kapil S. Meleveedu, Parikshit Padhi, Roberto Pili, Poolakkad S. Satheeshkumar

**Affiliations:** 1 ECMC Health Campus, 462 Grider St, University at Buffalo, Buffalo, NY 14215, USA; minuponn@buffalo.edu; 2 City of Hope Comprehensive Cancer Center, Duarte CA and Samuel Oschin Comprehensive Cancer Institute, Cedars-Sinai Medical System, Los Angeles, CA 91010, USA; jepstein@coh.org; 3 Carole and Ray Neag Comprehensive Cancer Center, University of Connecticut, Farmington, CT 06030, USA; kmeleveedu@uchc.edu; 4 Kaleida Health Infusion Center, 45 Spindrift Dr Suite 2000, University at Buffalo, Williamsville, NY 14221, USA; ppadhi@buffalo.edu; 5 Department of Medicine, Division of Hematology and Oncology, University at Buffalo, Buffalo, NY 14203, USA; rpili@buffalo.edu

**Keywords:** oral mucositis, chemotherapy, opioid, propensity score estimation, burden of illness, in-hospital mortality

## Abstract

**Objectives**: Mucositis is a debilitating side effect of cancer therapy that adversely affects quality of life, cost of care, and the outcome of cancer therapy. Oral mucositis-related pain may be treated with numerous modalities but often includes opioids. The effects of opiate treatment on painful UM and its overall influence on the burden of illness (BOI) in cancer patients remain unknown. **Methods:** This study utilized the 2017 United States (US) National Inpatient Sample (NIS) database. The exposure was opioid treatment for chemo-induced ulcerative mucositis (UM), oral mucositis-induced pain, and the main outcomes included in-hospital mortality and BOI, length of hospital stays (LOS), and total hospital charges. Multivariable regression analysis was used to examine the relationship between outcomes and the key independent variable, opioid use, adjusting for propensity scores. **Results**: In the propensity score-adjusted analysis, UM patients with opioid treatment had 0.51 times lower total charges (95% CI: 0.42–0.76) and 0.67 times shorter LOS (95% CI: 0.51–0.87) than the UM patients without opioid treatment. However, there was no association between opioid treatment and in-hospital mortality. In the sensitivity analysis, the effect estimates were comparable in the propensity score-adjusted analysis, the decile-adjusted model, and the full model with the non-propensity score estimated method. **Conclusions:** Cancer patients with chemotherapy-induced UM-prescribed opioid analgesics for treating pain are associated with a lower BOI. Opioid pain medications are commonly provided to cancer survivors; estimating the BOI among them is crucial in supportive care research.

## 1. Introduction

Adverse events of cancer treatment, such as mucositis affecting the oropharyngeal mucosa and other sites including the respiratory, gastrointestinal tract, and vaginal mucosa, may be associated with pain, bleeding, and discomfort and, when severe, may lead to modification or interruption in cancer therapy [1,2,3,4,5,6,7,8,9]. Given the importance of maintaining good oral health, individuals receiving cancer treatment may be given dental referrals before and during their course of treatment. Approaches to the management of mucositis include prevention of mucositis, including cryotherapy, keratinocyte growth factor-1 palifermin, low-level laser therapy (photobiomodulation therapy (PBM)), mucosal anesthetics, and analgesics, including topical interventions supported by research with good patient compliance [10,11,12,13,14,15,16,17] PBM has been shown to prevent mucosal injury, accelerate repair of mucosal injury, and provide analgesic effects. Despite the diverse information regarding mucositis outcomes, recent data indicate that the treatment of oral mucositis with topical morphine may offer no benefits beyond analgesia [18]. Furthermore, it is suggested that oral epithelial cells possess opioid receptors, and that morphine can enhance cell migration, both of which contribute to the healing process [10,11,12,13,14,15,16,17]. Additionally, patient-controlled analgesia with opioids is still commonly suggested for the management of pain associated with oral mucositis due to the severity of pain and challenges in pain management. Furthermore, cancer treatment-induced mucositis is associated with weight loss, systemic infection, longer hospital stays, and increased risk of morbidity and mortality [5,6,7,8]. An unpleasant side effect of cancer treatment, mucositis has a major negative influence on patients’ quality of life and contributes to a huge burden of illness by raising healthcare expenditures, delaying treatment, and increasing the risk of complications [3,4,5,6]. Open sores in the mucosa raise the risk of bacterial, fungal, and viral infections, which can be fatal, especially in patients with compromised immune systems [7,9]. Additionally, severe mucositis can cause treatment delays or reductions in cancer treatment, which may affect treatment efficacy and survival outcomes [1,2,3,4]. All of these factors are linked to increased resource use, including more frequent doctor visits, hospitalizations, and prolonged hospital stays. Physical problems, psychological distress, and financial strain can all have a substantial negative impact on one’s overall quality of life [8]. Therefore, our hypothesis is that treating mucositis lessens the impact on mental and physical health, lowers the risk of infections, lessens the financial burden of treatment and overall costs, and shortens hospital stays. In these instances, the impact of opioid management upon painful UM and the overall impact upon BOI is not known among cancer patients. To explore this impact, we utilized the United States National Inpatient Sample database to examine the impact of opioid use on cancer patients encountering UM.

## 2. Results

**Figure 1:**
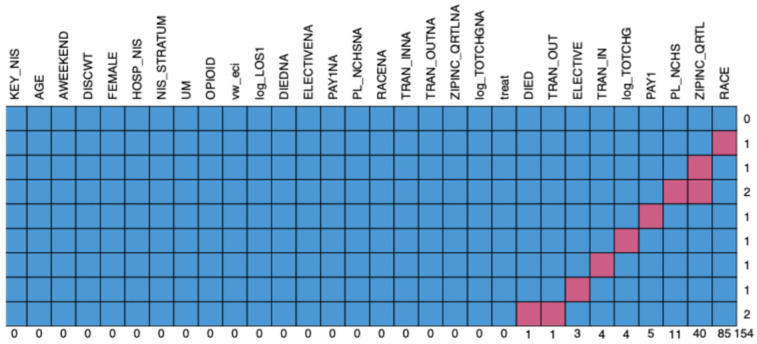
The plot of missing data patterns.

The percentage of missing data was 0.2%. Most of the missing values were race (3.6%), followed by median household income for the patient’s ZIP code (1.7%), patient location (0.5%), expected primary payer (0.2%), indicator of a transfer into the hospital (0.2%), total charge (0.2%), elective admissions (0.1%), transferred out (0.04%), and death (0.04%). The plot of missing data patterns revealed that data appeared to be missing at random throughout the dataset (Figure 1).

In the study, among 11,765 chemotherapy-induced UM patients, 365 (3.1%) were prescribed opioids (Table 1). Among the UM cohort, 27.6% received treatment for lymphoma, 21.8% received treatment for solid tumors with metastatic cancers, and 39.6% received treatment for solid tumors without metastasis. There was no difference in age, race, payer type, location type, or transfer type among UM patients with and without opioid treatment. UM with opioid treatment had higher comorbidity scores than the non-opioid treatment (16.8 vs. 14.1, *p* = 0.02). The LOS was 9.2 days for patients with UM and 5.9 days for those with opioid treatment. The total hospital charge for UM patients was USD 89,524, and USD 46,340 for those with opioid treatment. In-hospital mortality among patients with UM was 3.5% and 4.1% for those receiving opioid treatment.

**Figure 2:**
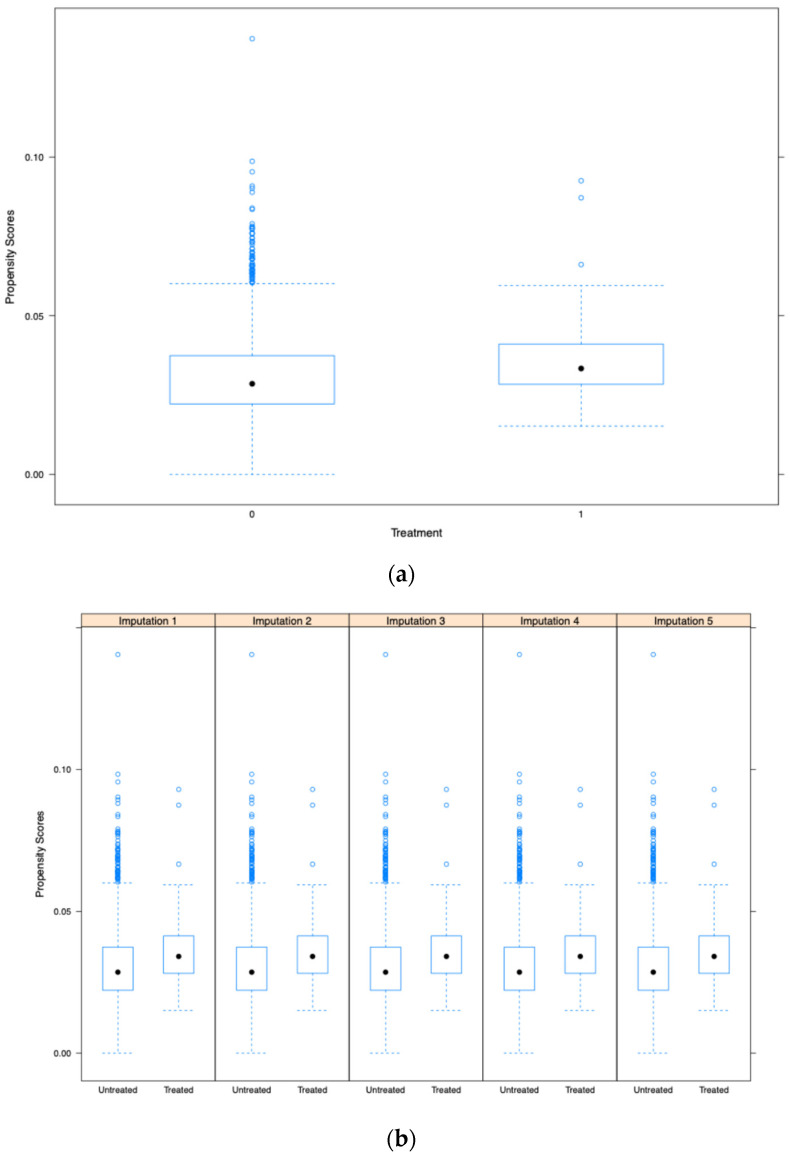
(**a**) The box–whiskers plot of the propensity score in the single dataset. (**b**) The box–whiskers plot of the propensity score in the partitioned dataset (five imputed datasets) for validation assessment.

**Figure 3:**
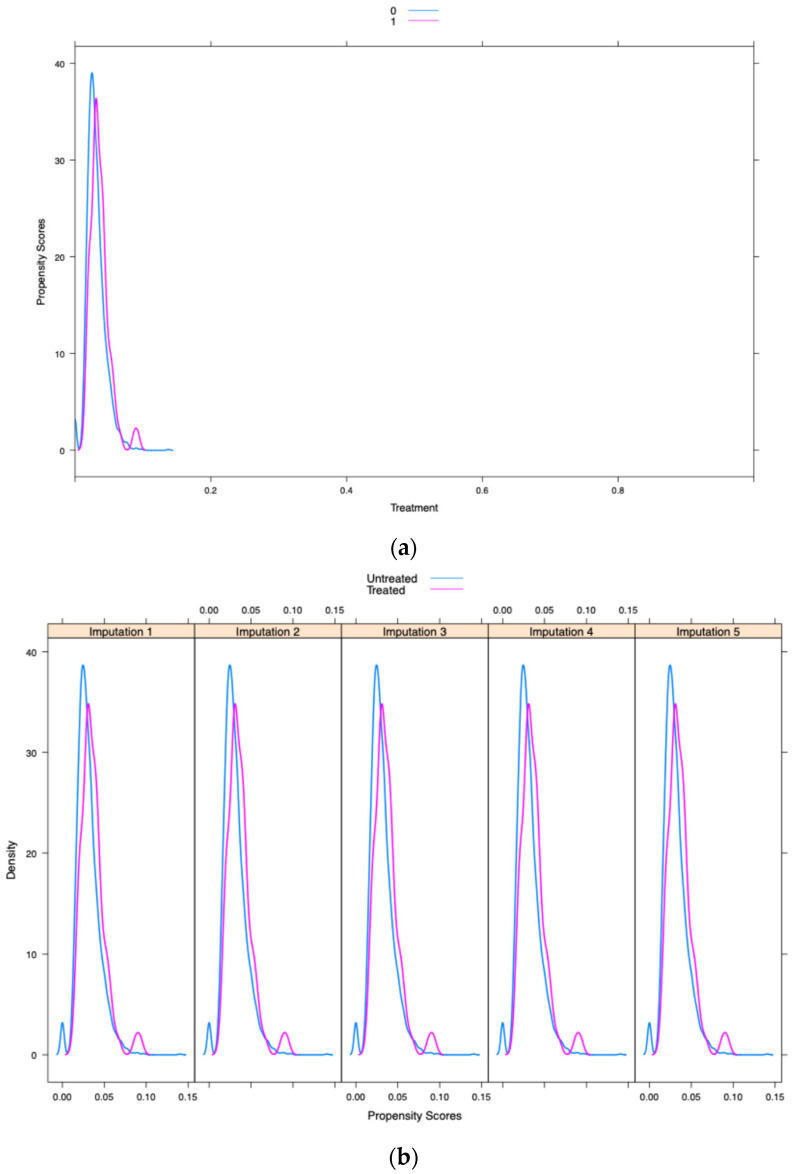
(**a**) The Kernel density plot of the propensity score in the single dataset. (**b**) The Kernel density plot of the propensity score from five imputed datasets.

**Figure 4:**
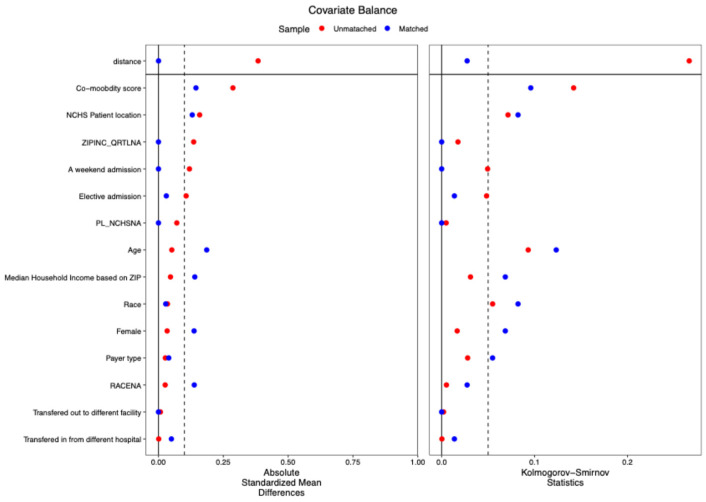
Covariate balance: the degree to which the distribution of covariates is similar across degrees of treatment is referred to as covariate balance. All measured variables were balanced between the groups.

**Figure 5:**
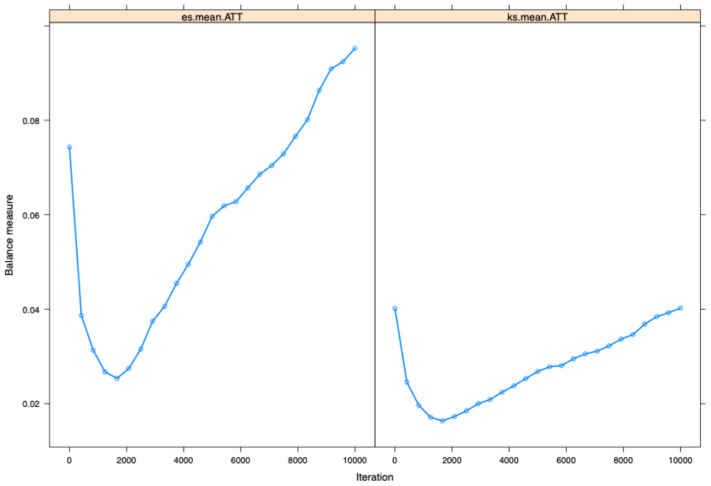
The maximum standardized mean differences between treatment (opioids) and non-treatment (non-opioids).

The common support of propensity score (overlap in the range of propensity scores between treatment and control groups is essential to ensure comparability of individuals for matching) distribution between the opioid and non-opioid treatment groups was evaluated visually using Kernel density and box and whiskers plots—this was performed in the main dataset and the multiple imputed datasets (Kernel density and box and whisker plots are employed to evaluate covariate balance post-propensity score matching, confirming the comparability of treatment and control groups for observed features). When the common support was assessed using Kernel density plots and box and whiskers plots, it was determined that it was sufficient to be incorporated into the study (Figure 2a,b and Figure 3a,b). Further, Figure 4 shows a love plot showcasing covariate balancing (love plots visually represent the pre- and post-effects of propensity scores). Figure 5 shows the maximum standardized mean differences between treatment (opioids) and non-treatment (non-opioids). Additionally, Table 2 shows propensity scores matched baseline characteristics of chemotherapy-induced ulcerative mucositis patients stratified with and without opioids, and further outcomes were assessed based on propensity score matched characteristics.

In the propensity score-adjusted analysis, UM patients with opioid treatment had 0.51 times lower total charges (95% CI: 0.42–0.76, *p* < 0.001) and 0.67 times lower LOS (95% CI: 0.51–0.87, *p* = 0.002) than the UM patients without opioid treatment. UM patients with opioid treatment had no difference in the in-hospital mortality compared to UM patients without opioid treatment. In the sensitivity analysis, the effect estimates were comparable in the propensity score-adjusted analysis, the decile-adjusted model, and the full model with the non-propensity score estimated method (Table 3).

The results of the subgroup analysis stratified to lymphomas showed that in the unadjusted analysis, the total charge (USD 59,889 vs. USD 115,697, *p* = 0.001) and LOS (6.36 days vs. 10.56 days, *p* = 0.01) were lower in the UM opioid group vs. the UM non-opioid group. However, there were no differences among solid tumors with metastasis between UM opioids and UM non-opioids for the outcome and total charge (USD 42,348 vs. USD 45,387, *p* = 0.69) and LOS (5.24 days vs. 5.58 days, *p* = 0.72). And in the solid tumors without metastasis between UM opioids and UM non-opioids for the outcome and the total charge (USD 35,364 vs. USD 41,476, *p* = 0.16) and LOS (4.79 days vs. 5.28 days, *p* = 0.41).

Further, we utilized the Elixir comorbidity score (adjusted in all analyses) and assessed the influence of these scores on covariate balancing, evaluated using the absolute standardized mean difference and Kolmogorov–Smirnov tests. Therefore, we assert that, after considering comorbidity status, we have analyzed the illness severity among the cohorts. Additionally, we examined the cohorts for the admission discharges, and there were no differences in the UM opioid vs. non-opioid use (Supplementary File). And the cancer cases included showed that most cancer cohorts treated with opioids for UM were solid cancers without metastasis (Supplementary File).

Patients with opioid use were slightly sicker compared to those without opioid use, as examined from the Elixir comorbidity score.

## 3. Discussion

The cohort of chemotherapy-induced UM patients represented cancer patients receiving treatment at various hospitals across the United States for treatment of solid tumors and lymphoma and an understudied population of patients with UM. With the Elixhauser comorbidity index status, we identified the cohort represented 23.8% receiving treatment for lymphoma, 27.2% receiving treatment for metastatic cancers, and 41.3% receiving treatment for solid tumors without metastasis. This study showed that cancer patients with chemotherapy-induced UM received 3.1% of the opioids. There was no difference in the baseline characteristics between the opioid treatment and non-treatment groups; however, patients with UM and treated with opioids were sicker with high comorbidity scores. Further, when treated with opioids for chemotherapy-induced UM, patients had lower total charges and LOS. This analysis is limited by a lack of clinical oral mucosal scoring of mucositis and patient-reported outcomes, including pain ratings. In the subgroup analysis, when stratified to the lymphoma group, the associations remained; however, when stratified to the solid tumor with and without metastasis, the association was not seen. These findings suggest that the best pain management practices may reduce BOI and LOS, leading to reduced cost of care.

UM is a common and severe consequence of cancer treatments, particularly chemotherapy and radiotherapy, characterized by inflammation, ulceration, and intense pain, leading to nutritional deficiencies, treatment delays, and a diminished quality of life [1]. The burden of illness associated with UM is substantial, affecting clinical outcomes, healthcare costs, and patient well-being. Clinically, UM increases the risk of localized and systemic infections, posing severe threats to immunocompromised patients [2]. The ulcerative lesions serve as entry points for opportunistic pathogens, heightening the risk of bacterial infections, including bacteremia and sepsis, commonly caused by Streptococcus, Staphylococcus, Escherichia coli, and Pseudomonas aeruginosa [3,7,9]. Fungal infections, particularly those caused by Candida albicans, often result in oral candidiasis and systemic fungal infections, while viral reactivations, such as herpes simplex virus (HSV), further exacerbate mucositis-related pain and delay healing [1,7,9]. The severity of UM is directly linked to increased hospitalization rates, prolonged ICU admissions, and the necessity for broad-spectrum antimicrobial therapies, leading to soaring healthcare costs, with estimated expenses per patient reaching up to USD 25,000 due to infection-related complications and supportive care requirements [7,8]. Additionally, UM significantly impacts patients’ well-being, contributing to malnutrition, weight loss, psychological distress, and reduced adherence to cancer treatment, further compromising therapeutic outcomes [9,10]. The economic burden extends beyond direct medical costs to indirect costs, including loss of productivity and caregiver strain. Various management strategies have been implemented to reduce the burden of UM, including preventive oral care with antimicrobial mouth rinses, antimicrobial prophylaxis, PBM, and biological agents such as palifermin, which promotes mucosal healing and decreases ulceration risks [11,12]. Given its profound clinical, economic, and psychological burden, UM remains a significant challenge in oncology, necessitating effective prevention and treatment approaches to mitigate its impact and improve patient outcomes [10,11].

Research demonstrates that effective treatment of UM leads to substantial enhancements in clinical outcomes, quality of life, and overall healthcare efficiency for patients. Alleviation of pain and restoration of mucosal integrity enable patients to sustain enough nutrition and hydration, hence diminishing the likelihood of malnutrition and weight loss, which are prevalent consequences of severe oral mucositis [8]. Effective treatments also reduce the risk of secondary infections, including bacterial, fungal, and viral problems, hence diminishing the chance of sepsis and the necessity for hospitalization or intense antimicrobial therapy [2]. By mitigating the severity and length of UM, patients can more reliably adhere to their cancer treatment protocols, thereby averting dose reductions or treatment pauses that may jeopardize therapeutic efficacy and overall prognosis [3]. In the present investigation, the lack of data pertaining to infectious complications precluded us from presenting the incremental illness occurrences unique to mucositis. Consequently, the analysis of the BOI proxies—costs and duration of stay—yields comprehensive insights about healthcare utilization and the disease burden. Moreover, efficient operations management significantly decreases healthcare expenses by reducing hospital admissions, intensive care unit stays, and the reliance on expensive supportive care measures such as complete parenteral feeding [4]. Patients exhibit less distress, anxiety, and despair when mucositis-related pain is effectively managed, enhancing their overall well-being and capacity to participate in everyday activities [5]. Advanced therapeutic techniques, such as low-level laser therapy (LLLT or PBM), palifermin, cryotherapy, and enhanced oral hygiene procedures, have demonstrated considerable efficacy in mitigating oral mucositis symptoms and averting serious consequences [6]. The successful management of UM not only improves patient comfort and therapeutic adherence but also leads to enhanced cancer treatment outcomes, increased survival rates, and a diminished strain on healthcare systems [7].

We strongly believe that the application of propensity score-based analysis is an effective statistical technique employed in observational studies to mitigate bias and confounding in evaluating the burden of illness in patients with UM. This methodology enables researchers to juxtapose patients with UM against a comparable cohort devoid of UM by equilibrating confounders such as age, comorbidities, cancer type, treatment regimen, and other confounding variables that may affect health outcomes. A principal use of propensity score matching in operations management research is the assessment of healthcare utilization and expenditures. Through the comparison of matched cohorts, researchers can precisely quantify the supplementary economic burden linked to UM, encompassing elevated hospitalization rates, intensive care admissions, extended length of stay, and the expenses of supportive care interventions such as parenteral nutrition, analgesics, and antimicrobial therapies [2]. This strategy distinguishes the direct effects of UM from other underlying disorders that may affect healthcare costs. Moreover, propensity score-based analysis is essential in evaluating clinical outcomes and treatment delays. Patients with UM frequently necessitate dosage adjustments, therapy pauses, or alternate medications due to intense pain, infections, or nutritional inadequacies. By comparing individuals with and without UM, researchers can ascertain the impact of mucositis on treatment adherence, cancer development, and survival rates.

Moreover, propensity score methodologies facilitate the quantification of the influence of UM on quality of life (QoL) by reducing confounding in the analysis of patient-reported outcomes concerning pain, functional limitations, psychological distress, and social isolation. This allows researchers to produce substantial evidence regarding the impact of mucositis on daily activities, emotional health, and long-term healing. Employing propensity score matching, weighting, or stratification enables studies to yield causal insights on the actual burden of illness in UM patients within real-world contexts, hence enhancing the validity of results and guiding focused therapies. This method finally facilitates the development of economical treatment methods, enhances supportive care protocols, and improves health outcomes for cancer patients suffering from UM.

We have included distinct information for the cohorts with admission discharges and cancer cohorts and found no difference in UM opioid versus non-opioid utilization (Table S1 Supplementary File). The cancer cases indicated that the majority of cancer cohorts administered opioids for UM were solid tumors without metastasis. We could not evaluate the source of the pain and its intensity in these patients. In all our analyses, we controlled for transfers out of the institution to investigate the relationship between cost and duration of stay; however, no associations were found in either the unadjusted or adjusted analyses. Figure 4 illustrates the covariate balancing concerning the transferred-out that may influence the evaluated outcomes. Had there been a substantial disparity in the unadjusted analysis presented in Table 1, we would have pursued classification depending on whether subjects were transferred out or not.

Our novel analytics–propensity score (PS) estimated analysis in our study of big data for adverse events in oncology care. Although PS estimated analysis is increasingly used, there is a dearth of this analysis, particularly in supportive care cancer settings with adverse event estimation. Utilizing observational studies and big data with the PS estimated analysis might support improved understanding of real-world scenarios [19,20,21,22]. The traditional method for adjusting for baseline variations between treatment groups is covariate adjustment. All relevant patient characteristics are included in a regression model involving the result of interest to the alternative treatments. However, concerns about overfitting are frequently expressed when the number of variables is enormous compared to the number of patients or outcome events [23].

The purpose of the PS method is to mitigate the confounding effects and intervention allocation. A PS technique is described as the chance of a patient receiving an intervention given a collection of factors; simultaneously, PS shrinks all patient features into a single covariate [24,25]. There are many PS estimation methods utilized in studies, including stratification, matching, weighting, and adjusting for PS as a covariate [26,27]; we utilized matching and adjusting for the PS as a covariate in the regression model. There is, however, a dearth of instruction on how to make an informed choice between these numerous PS approaches or conventional covariate adjustment [28]. Thus, we used various PS approaches on our large-scale observational NIS datasets to evaluate each method’s unique advantages and disadvantages and compare their results to those obtained using conventional covariate adjustment [29]. Our results showed that all the methods generated comparable effect estimates.

The study cohort was stratified into five strata based on the propensity scores; this method removes 90% of the bias owing to measured confounders. We assessed the variances through the common support evaluation and the box and whiskers plot. It was found that the common support was sufficient to be included in the study after it was examined using Kernel density plots and box and whiskers plots. The balance property and the common support region are two fundamental aspects of propensity score models used to examine their adequacy and underlying assumptions [30]. The balance attribute here reflects the covariates’ means and variances (potentially on the interaction and polynomial terms). The common support region describes the overlap of two propensity score distributions.

In contrast, evidence shows reduced survival and progression-free survival among cancer patients with opioid users [31,32]. However, patients diagnosed with pancreatic cancer who are given opioid treatment have a better chance of surviving the disease for a longer period of time. Findings are complex when understanding how the pain management, molecular and systemic factors, and access to opioids affect the burden of illness and mortality among cancer patients who are undergoing advanced care [33].

The treatment of mucositis is still built on the foundation of topical anesthetics and, in more severe instances, the use of analgesics, particularly opioids, that are administered systemically [34]. It would be advantageous to reduce the unpleasant symptoms that are associated with opioid use, such as nausea, vomiting, neurologic symptoms, constipation, and drowsiness, with the help of local opioid administration [35]. In addition, it has been demonstrated on several occasions both in vitro and in vivo that opioids have an effect on the proliferation and survival of cells [36,37]. Opioids, due to their capacity to stimulate the migration of keratinocytes and their role in inflammation, have been shown to speed the healing of wounds and the re-epithelialization of skin wounds [14,38,39,40], which may represent one of the benefits of using opioids in the management of painful UM.

Approximately 80% of liquid cancer patients receiving a conditioning regimen for transplants suffer from UM accompanied by intraoral pain, bleeding, difficulty with eating and drinking, communication, etc. [41]. Following a review of the adequacy of medication used to treat UM, drug-based interventions, including opioids, were successfully employed to treat UM [42,43]; however, estimating its efficacy on clinical outcomes is seldom investigated, such as the burden of illness and mortality. According to pain management principles, opioids can successfully treat 80–90% of cancer-related pain, thus improving crucial components for improving patients’ clinical results and quality of life [42,43,44].

Due to the fact that these symptoms make it difficult to ingest following treatment, weight loss and a slowed recovery may occur. Therefore, there is a strong correlation between treatment efficacy, active pain management, clinical outcomes, and the burden of illness and mortality [45]. When cancer pain was effectively addressed with the optimal dose of opioid analgesics and adhered to recommendations, a greater number of patients received the necessary care for their discomfort in less time [46].

We validated our findings when comparing solid and liquid cancers and thus increased the validity of the findings. The retrospective nature of the dataset, lack of longitudinal investigation, and monitoring of pain were challenging. Therefore, testing was limited to a small subset of the UM patients receiving opioids for treatment. We were unable to identify the causes of moderate to severe pain in cancer patients; the hypothesis was driven to investigate the use of opioid analgesics for UM and evaluate the appropriateness of opioid analgesic use despite these limitations. The dataset’s uniqueness points to the judiciousness of assessing the outcome analyzed through the total charges for those receiving opioids for pain management. Our earlier work approximated [7,9] the total charges for UM among patients receiving liquid and stem cell transplants, and as a result, the costs incurred for receiving opioid treatment were computed.

Additionally, the outcomes of interest studied are not specifically pain-related outcomes; the rationale behind anticipating an effect of opioid therapy on LOS and/or total charges, such as improved pain management resulting in shorter stays, mucositis is associated with higher morbidity and mortality [47], and systemic infections such as febrile neutropenia [9]. For those with advanced cancer, there may be a stronger link between the disease’s progression and opioid use. It is crucial to continue using opioids for efficient pain management since improvements in quality of life and function may be attained if cancer pain is successfully managed. As we showed in our previous studies, the relationship between outcomes differs for solid tumors vs. liquid tumors [7,9] in burden of illness (LOS and cost) and mortality. In this study, we assessed those variations, particularly when opioids were prescribed.

### Limitations

Our big data collection allowed us to extrapolate opiate use among cancer patients nationwide. The study findings should be interpreted in the context of the study limitations. Study limitations that include the following. Limited information to assess the prescription’s suitability for specific claims or ulcerative mucositis-related discomfort. The research relied on dispensing claims data, which may have excluded individuals who refused to take opioids. We examined cancer patients’ opioid claims. Unfortunately, it is unclear if these claims are connected to cancer pain management and assess the status of pain origin or its severity. The classification of ulcerative mucositis using ICD-10-CM (K1231) may insufficiently represent variations in disease severity. Given the premise of severity status potentially influencing the result, we have subsequently adjusted the comorbidity status in all forms of adjusted analysis. To this end, we employed the Elixir comorbidity score and assessed its influence on covariate balancing, as evaluated using the absolute standardized mean difference and the Kolmogorov–Smirnov tests. However, in the absence of precise severity status, this may impact treatment decisions and outcomes, necessitating categorization based on severity when such data are unavailable. Consequently, this may influence the interpretation of the data; however, we assert that by including comorbidity status, we have mitigated some of these constraints. NIS data are discharges; thus, it is possible for a patient to be readmitted and counted more than once, though the likelihood is small. We did not examine the true cost of care; rather, we used hospital charges, which could be different across different hospitals. Although we adjusted for confounders in the study, there could be other variables associated with opioid use, which we were not able to include in our study due to limitations of the dataset.

## 4. Methods

### 4.1. Study Design and Data Source

We utilized the United States 2017 National Inpatient Sample database, obtained from the Healthcare Cost and Utilization Project (HCUP) of the Agency for Healthcare Research and Quality. The NIS dataset comprises patient sociodemographic and comorbidity information, in-hospital outcomes, hospital characteristics, and hospitalization charges [48]. Our study used ICD-10-CM billable codes to identify chemotherapy-induced UM (K1231).

### 4.2. Study Measurements

The main exposure of this study was opioid use (yes/no), which was assessed using ICD-10-CM code (Z798891). The outcome variables were hospital length of stays (LOS), total charges, and in-hospital mortality. The LOS measured in days was calculated by subtracting the admission date from the discharge date. Total charges included the total charge of the health services (in USD)—it includes all hospital utilization fees charged by the hospital but excludes physician payments. In-hospital mortality was defined as mortality that happened during hospitalization coded from discharge disposition of patient (alive or dead).

Covariates included patient-level and clinical-level characteristics. Patient characteristics included in the study were age, sex (male or female), race (White, Black, Hispanic, Asian, and others), primary payer (Medicare, Medicaid, private insurance, and others), median household income based on ZIP code (first to the fourth quartile), and patient’s location (urban/rural—using a six-category urban–rural classification scheme for the United States’ counties developed by the National Center for Health Statistics). Clinical characteristics included admission origin (transferred-in and not-transferred), transfer type (an indicator of a transfer out of the hospital), admission type (elective vs. non-elective; elective indicates whether patients were electively hospitalized), and Elixhauser comorbidity index, which was used to categorize comorbidities. The Elixhauser comorbidity index variables are listed in the HCUP database [49].

### 4.3. Statistical Analysis

For the main analysis, chemotherapy-induced UM and the outcomes, LOS, total charges, in-hospital mortality, and opioid use as independent variables, different propensity score estimated analyses were computed, and the results were compared to the multivariable regression analysis [50,51]. For these investigations, it is essential to compute missing values in the NIS data; we employed multiple imputations as advised by the HCUP. The missing data pattern plot was used to determine whether there was a regular pattern in our data and to determine the randomness of the missing values. Five imputed datasets were produced, and we ran the analysis on each of them several times before combining the results of the five datasets. As the outcomes total charges and LOS were not normally distributed, we log-transformed, and the geometric mean (the log-transformed values were transformed back) was presented. A value of 0.0001 was imputed for LOS of 0 days to avoid negative log values. We then estimated propensity scores by applying logistic regression models on all five imputed datasets to estimate the log odds of the probability of having opioids based on possible confounders—age, sex, payer type, patient location, race, elective admission, an indicator of a transfer into or out of the hospital, median household income, comorbidity score, and whether metastatic cancer or not, whether solid tumor or not, and whether lymphoma or not. The use of observational studies—also known as nonrandomized studies—to gauge how treatments affect outcomes is growing in favor. In observational studies, the treatments are often chosen based on the characteristics of the individuals themselves. Because of this, the baseline characteristics of treated participants and untreated hospitalized cancer patients often differ in a systematic way. The systematic differences in baseline characteristics between treated and untreated participants must therefore be taken into account when evaluating the impact of treatment on outcomes. In order to estimate treatment effects from observational data, confounding must be reduced or eliminated. Further, we aim to familiarize the readers with the propensity score-based estimation in a cancer-based supportive/palliative setting and explain how methods based on it may be used to achieve this goal—through comparison of effect estimates by propensity score and by the regression-based methods. Further, all effect estimates were transformed back to the antilog scale.

The utility of propensity score estimation for large data in the toxicity of cancer therapies is less often observed. Hence, to evaluate whether the distribution of propensity scores across treatment (opioids) and comparison groups (non-opioids) is balanced or not, it is necessary to guarantee that the range of propensity scores across treatment and comparison groups overlaps (this is referred to as “common support” in statistical terms). When a treated individual is not comparable to an individual with a similar propensity score, it is impossible to draw any conclusions about the treatment’s impact. We utilized the common support evaluation with a box and whisker plot and a Kernel density plot to evaluate this. Additionally, a subgroup analysis was performed stratified to solid non-metastatic and metastatic cancers and lymphomas.

After the propensity score was estimated, we fitted the generalized linear models for assessing the association between opioid status and the outcomes (LOS, total charges, and in-hospital mortality), adjusting for the propensity score in the model. A sensitivity analysis was performed on propensity score analysis with deciles and compared to the regression analysis adjusting for the propensity score; correspondingly, the non-propensity score estimation analysis was computed using the full adjusted regression-generalized linear model. All statistical analyses were two-tailed, and statistical significance was determined using *p* < 0.05 and was performed using R 3.6.1 (R Foundation for Statistical Computing, Vienna, Austria).

## 5. Conclusions

Our findings indicate that opioid treatment for UM enhances BOI, suggesting that characteristics associated with increased resource utilization, such as more frequent medical consultations, hospitalizations, and extended hospital stays, may be influenced. The resource utilizations for health issues, psychological discomfort, and financial strain can significantly influence outcomes resulting from the treatment of UM. Consequently, our hypothesis remains valid: treating mucositis reduces the total burden of illness, diminishes expenses, and shortens hospital stays. Our results were supported by various propensity score-adjusted models and regression models, demonstrating that opioid drugs given to cancer patients for pain relief during UM are linked to a decreased BOI.

The application of various propensity score adjusted models in cancer supportive care may suggest causal relationships. Consequently, propensity score techniques can be utilized, such as any other statistical tool; however, they should be employed only when they are likely to yield advantageous outcomes.

## Figures and Tables

**Table 1:** Baseline characteristics of chemotherapy induced-ulcerative mucositis patients with and without opioid use.

	Ulcerative Mucositis Without Opioid Use (Survey Weighted)	Ulcerative Mucositis with Opioid Use (Survey Weighted)	*p*-Value
*n*	11,400 (96.9%)	365 (3.1%)	
Age (mean (SD))	56.88 (15.9)	56.14 (14.4)	0.64
Sex (%)			0.79
Female	5900.0 (51.8)	195.0 (53.4)	
Race (%)			0.08
White	7665.0 (69.7)	220.0 (62.9)	
Black	1170.0 (10.6)	70.0 (20.0)	
Hispanic	1200.0 (10.9)	25.0 (7.1)	
Asian and others	955.0 (8.7)	35.0 (10.0)	
Median household income (based on current year)			0.94
0–25th percentile	2445.0 (21.8)	70.0 (19.2)	
26th to 50th percentile	2840.0 (25.4)	95.0 (26.0)	
51st to 75th percentile	2960.0 (26.4)	105.0 (28.8)	
76th to 100th percentile	2955.0 (26.4)	95.0 (26.0)	
Expected primary payer (%)			0.89
Medicare	4205.0 (37.0)	145.0 (39.7)	
Medicaid	1570.0 (13.8)	45.0 (12.3)	
Private insurance	4980.0 (43.8)	150.0 (41.1)	
Self-pay, no charge, and other	620.0 (5.5)	25.0 (6.8)	
Patient location: NCHS urban–rural code (%)			0.36
“Central” counties of metro areas of >=1 million population	3615.0 (31.9)	90.0 (24.7)	
“Fringe” counties of metro areas of >=1 million population	2930.0 (25.8)	105.0 (28.8)	
Counties in metro areas of 250,000–999,999 population	2335.0 (20.6)	65.0 (17.8)	
Counties in metro areas of 50,000–249,999 population and micropolitan counties and not metropolitan or micropolitan counties	2465.0 (21.7)	105.0 (28.8)	
Admission type (%)			0.40
Elective	3830.0 (33.6)	105.0 (28.8)	
Indicator of a transfer out of the hospital			0.95
Transferred out	960.0 (8.4)	30.0 (8.2)	
Weighted Elixir score mean (SD)	14.13 (9.48)	16.79 (9.29)	0.02
Length of stay (geometric mean)	9.1 days	5.9 days	<0.001
Total charge (geometric mean)	USD 89,524	USD 46,340	<0.001
Mortality (%)	395.0 (3.5)	15.0 (4.1)	0.76
Lymphoma (%)	3135.0 (27.5)	110.0 (30.1)	0.63
Solid tumors with metastatic cancers (%)	2460.0 (21.6)	110.0 (30.1)	0.12
Solid tumors without metastatic cancers (%)	4450.0 (39.0)	210.0 (57.5)	0.002

Abbreviations: SD, standard deviation; NCHS, National Center for Health Statistics; USD, United States Dollar. Note: All frequencies and percentages are weighted.

**Table 2:** Propensity scores matched baseline characteristics of chemotherapy-induced ulcerative mucositis patients stratified by with and without opioids; outcomes were assessed based on propensity score-matched characteristics.

	Ulcerative Mucositis Without Opioid Use (Weighted)	Ulcerative Mucositis with Opioid Use (Weighted)	*p*-Value
*n*	73	73	
Age (mean (SD))	55.3 (16.7)	56.14 (14.4)	0.75
Sex (%)			0.74
Female	42.0 (57.8)	39.0 (53.4)	
Race (%)			0.17
White	47.0 (64.4)	46.0 (63.0)	
Black	26.0 (35.6)	15.0 (36.9)	
Median household income (based on current year)			0.79
0–25th percentile	14.0 (19.2)	14.0 (19.2)	
26th to 50th percentile	24.0 (32.9)	19.0 (26.0)	
51st to 75th percentile	17.0 (23.3)	21.0 (28.8)	
76th to 100th percentile	18.0 (24.7)	19.0 (26.0)	
Expected primary payer (%)			0.77
Medicare and Medicaid	50.0 (56.1)	47.0 (42.0)	
Others	29.0 (44.8)	30.0 (47.9)	
Patient location: NCHS urban–rural code (%)			0.75
“Central” counties of metro areas of >=1 million population	22.0 (30.1)	18.0 (24.7)	
“Fringe” counties of metro areas of >=1 million population	23.0 (31.5)	21.0 (28.8)	
Counties in metro areas of 250,000–999,999 population	12.0 (16.4)	13.0 (17.8)	
Counties in metro areas of 50,000–249,999 population and micropolitan counties and not metropolitan or micropolitan counties	16.0 (21.9)	21.0 (28.8)	
Admission type (%)			0.85
Elective	23.0 (31.5)	21.0 (28.8)	
Weighted Elixir score mean (SD)	17.52 (8.38)	16.79 (9.29)	0.62
Length of stay (geometric mean)	9.1 days	5.9 days	0.02
Total charge (geometric mean)	USD 76,863	USD 46,341	0.004

Abbreviations: SD, standard deviation; NCHS, National Center for Health Statistics; USD, United States Dollar. Note: All frequencies and percentages are weighted.

**Table 3:** Sensitivity analysis (comparison of the outcomes for different propensity score-adjusted analyses).

Effect Estimates	Propensity Score-Adjusted Model ^1^	Propensity Score with a Deciles-Adjusted Model ^2^	Fully Adjusted Model (Non-Propensity Score Estimation) ^3^
Total charge	0.51 (0.42–0.76), *p* < 0.001	0.57 (0.42–0.76), *p* < 0.001	0.56 (0.44–0.71), *p* < 0.001
Length of stay	0.67 (0.51–0.87), *p* = 0.002	0.67 (0.51–0.87), *p* = 0.003	0.67 (0.54–0.82), *p* = 0.0002
In-hospital mortality (aOR)	0.87 (0.21–2.41), *p* = 0.81	0.85 (0.20–2.39), *p* = 0.79	0.93 (0.31–2.82), *p* = 0.90

^1.^ Propensity score-adjusted model—after calculating the propensity score, there are numerous ways to use it in an analysis. The first approach we used was to control for the propensity score using traditional regression generalized linear model approaches. ^2.^ Propensity scores with a deciles-adjusted model—stratifying the subjects based on the propensity score quintiles or deciles is an option for controlling for the propensity score. The deciles of the propensity scores were used to stratify the participants. ^3.^ Effect estimates of the fully adjusted model (non-propensity score estimation)—a regression analysis with a generalized linear model adjusting for the full covariates.

## Data Availability

We used data from the 2017 NIS database obtained from the Healthcare Cost and Utilization Project (HCUP) of the Agency for Healthcare Research and Quality (AHRQ). International Classification of Diseases, Tenth Revision, Clinical Modification (ICD-10-CM) provided in manuscript. All statistical analyses were performed using R, Version 3.6.2 (R Foundation for Statistical Computing, Vienna, Austria).

## Supplementary Materials

The following supporting information can be downloaded at: https://www.mdpi.com/article/10.3390/ph18040536/s1, Table S1: Discharge disposition of ulcerative mucositis with and without opioid use.
