# Supplementary material for: Hospitalized Cancer Patients with Opioid Management for Chemo-Induced Ulcerative Mucositis Lessens the Patients’ Overall Burden of Illness
Source: Pharmaceuticals (Basel). 2025 Apr 6;18(4):536. doi: 10.3390/ph18040536 (PMC12030479; doi:10.3390/ph18040536)
Supplement: Supplementary file 1 [file pharmaceuticals-18-00536-s001.zip › pharmaceuticals-3507504-supplementary.pdf]

Table S1. Supplementary file

Discharge disposition of ulcerative mucositis with and without opioid use

Discharge Disposition of Patient (No, %)	Ulcerative mucositis without Opioid use (Weighted)	Ulcerative mucositis with Opioid use (Weighted)
Discharge to home/self-care	7340.0 (64.4)	235.0 (64.4)
Discharged/transferred to a Short-Term General Hospital for Inpatient Care	110.0 (1.0)	
Discharged/transferred to another type of institution not defined elsewhere	850.0 (7.5)	30.0 (8.2)
Discharged/transferred to Home under care of Organized Home Health Service Organization in anticipation of covered skilled care	2660.0 (23.3)	85.0 (23.3)
Against medical advice	40.0 (0.4)	
Dead	395.0 (3.5)	15.0 (4.1)
